# Gastric cancer-derived mesenchymal stem cells prompt gastric cancer progression through secretion of interleukin-8

**DOI:** 10.1186/s13046-015-0172-3

**Published:** 2015-05-20

**Authors:** Wei Li, Ying Zhou, Jin Yang, Xu Zhang, Huanhuan Zhang, Ting Zhang, Shaolin Zhao, Ping Zheng, Juan Huo, Huiyi Wu

**Affiliations:** Center of Research Laboratory, The First People’s Hospital of Lianyungang, 182 Tongguan Road, Lianyungang, 222001 China; School of Medical Science and Laboratory Medicine, Jiangsu University, 301 Xuefu Road, Zhenjiang, 212013 China; Department of Pathology, Xuzhou Medical College, 209 Tongshan Road, Xuzhou, 221004 China

**Keywords:** Mesenchymal stem cells, Gastric cancer, Interleukin-8, Proliferation, Migration, Angiogenesis

## Abstract

**Background:**

Bone marrow mesenchymal stem cells (BM-MSCs) have been identified to be closely associated with tumor growth and progression. However, the roles of tumor-resident MSCs in cancer have not been thoroughly clarified. This study was to investigate the regulating effect of gastric cancer-derived MSCs (GC-MSCs) on gastric cancer and elucidate the underlying mechanism.

**Methods:**

GC-MSCs were isolated from primary human gastric cancer tissues and characterized. The effect of GC-MSCs on gastric cancer cell proliferation was analyzed by MTT assay and colony formation assay. Transwell migration assay was performed to evaluate the influence of GC-MSCs in gastric cancer cell migration. The regulating effects of interactions between gastric cancer cells and GC-MSCs on their pro-angiogenic abilities were analyzed in a co-culture system, with the expression, and secretion of pro-angiogenic factors detected by RT-PCR and Luminex assay. Tube formation assay was used to further validate the angiogenic capability of gastric cancer cells or GC-MSCs. Cytokine profiles in the supernatant of GC-MSCs were screened by Luminex assay and neutralizing antibody was used to identify the key effective cytokines. The activations of Akt and Erk1/2 in gastric caner cells were detected by Western blot.

**Results:**

GC-MSC treatment enhanced the proliferation and migration of BGC-823 and MKN-28 cells, which was more potently than MSCs from adjacent non-cancerous tissues (GCN-MSCs) or bone marrow (BM-MSCs). Higher expression levels of pro-angiogenic factors were detected in GC-MSCs than GCN-MSCs or BM-MSCs. After 10 % GC-MSC-CM treatment, BGC-823, and MKN-28 cells expressed increased levels of pro-angiogenic factors and facilitated tube formation more potently than cancer cells alone. Furthermore, GC-MSCs produced an extremely higher level of interleukin-8 (IL-8) than GCN-MSCs or BM-MSCs. Blockade of IL-8 by neutralizing antibody significantly attenuated the tumor-promoting effect of GC-MSCs. In addition, 10 % CM of IL-8-secreted GC-MSCs induced the activations of Akt or Erk1/2 pathway in BGC-823 and MKN-28 cells.

**Conclusion:**

Tumor-resident GC-MSCs promote gastric cancer growth and progression more efficiently than GCN-MSCs or BM-MSCs through a considerable secretion of IL-8, which could be a possible target for gastric cancer therapy.

## Background

Gastric cancer is one of the most frequent malignant tumors and a leading cause of cancer death worldwide [1]. Although recent improvements have resulted in a decrease in the mortality of gastric cancer, it remains a serious health problem with poor prognosis [2–4]. Deficiency of targeted agents seems to restrict the therapeutic strategy for gastric cancer and a thorough understanding of the mechanism is urgently needed.

Tumor microenvironment (TME) has been indicated to play a critical role in both the initiation and progression of tumors [5–7]. Beside of soluble molecules, TME is composed of various kinds of cells, such as fibroblasts, tumor-associated macrophages, and endothelial cells [8]. Interactions between tumor cells and the surrounding TME contribute to the growth and metastasis of tumors [9, 10]. Thus, better understanding of the association between gastric cancer cells and components in TME could be directory for elucidating the underlying mechanisms of gastric cancer.

As one of the key components in TME, mesenchymal stem cells (MSCs) have attracted much attention and have been demonstrated to affect the fate of tumor cells in several reports [11–13]. A growing body of evidence indicates that MSCs is closely associated with the initiation, growth, and metastasis of various types of tumors. It has been demonstrated that bone marrow-derived MSCs (BM-MSCs) are capable to promote growth and angiogenesis in both breast and prostate tumors through the crosstalk between BM-MSCs and tumor cells [14]. Another report indicated that lymphoma-derived MSCs could promote tumor development by recruiting monocytes into the inflammatory TME [15]. In addition, gastric submucosa-resident mesenchymal stem cells have been shown to contribute to cancer stroma formation and play an important role in gastric cancer progression [16]. However, the potential roles of gastric cancer-derived MSCs (GC-MSCs) in gastric cancer and the exact mechanisms have not been thoroughly investigated.

As a multifunctional pro-inflammatory cytokine, interleukin-8 (IL-8) could increase cancer cells proliferation and migration [17, 18], stimulate tumor angiogenesis [19] and induce epithelial-mesenchymal transition of cancer cells [20]. The level of IL-8 has been demonstrated to be indicative of poor prognosis in gastric cancer [21]. However, the underlying mechanisms of IL-8-medicated gastric cancer progression are still obscure. Recently, the role of IL-8 in the interactions between gastric cancer cells and MSCs has drawn much interest. Kidney cancer cell secreted IL-8 has been demonstrated to promote migration of BM-MSCs by activating the Akt signaling pathway [22]. Another report indicated that IL-8 could enhance the angiogenic potential of human BM-MSCs by stimulating VEGF production [23]. Nevertheless, the role of IL-8 in the regulating effect of tumor resident-MSCs on gastric cancer cells has not been studied.

In this study, we isolated GC-MSCs from primary human gastric cancer tissues, characterized their phenotype, and studied their effect on gastric cancer growth and progression in comparison to adjacent non-cancerous tissues-derived MSCs (GCN-MSCs) and BM-MSCs. Furthermore, molecular mechanisms involved in the tumor-promoting effect of GC-MSCs were also investigated.

## Materials and methods

### Cells

GC-MSCs were isolated as described previously [24] with a minor modification. Gastric cancer tissues were obtained from patients who underwent radical gastrectomy in the First People’s Hospital of Lianyungang (Jiangsu, China), and the procedure was approved by the Ethics Committee of the First People’s Hospital of Lianyungang. Briefly, fresh tumor tissues were collected and washed off the blood. After rinsed in antibiotics to avoid contamination, the tissue specimens were cut into 1-mm^3^-sized pieces and placed directly into culture dishes for 30 min to improve adhesion. Then, the tissue explants were floated in growing medium of L-DMEM (Gibco, Invitrogen Corporation, Carlsbad, CA, USA) containing 15 % (v/v) fetal bovine serum (FBS; Gibco), penicillin (100 U/ml), and streptomycin (100 μg/ml), and subsequently incubated at 37 °C in humid air with 5 % CO_2_. When the fibroblast-like cells reached subconfluence, tissue pieces were removed, and adherent cells were digested and passaged into flasks for further expansion. At 4 to 5 passages, a homogeneous cell population was obtained and used for the subsequent experiments. Paired adjacent non-cancerous tissues located more than 5-cm away from the tumor site were collected from the same patients for GCN-MSCs isolation, which was performed in a similar manner to GC-MSCs. GCN-MSCs and bone marrow mesenchymal stem cells (BM-MSCs) were chosen as the controls.

Human gastric cancer cell lines of BGC-823 and MKN-28 were gifts from the First Affiliated Hospital of Soochow University (Jiangsu, China). Cells were cultured in RPMI 1640 medium (Gibco) supplemented with 10 % (v/v) FBS in 5 % CO_2_ humidified atmosphere at 37 °C.

### Osteogenic and adipogenic differentiation

GC-MSCs (passage 4) were seeded in a 6-well plate at 3 × 10^4^ cells/cm^2^ and cultured in L-DMEM containing 15 % FBS. For osteogenic differentiation, GC-MSCs were cultured in human mesenchymal stem cell osteogenic differentiation medium (Cyagen Biosciences, Sunnyvale, CA, USA) for 14 days and stained with alizarin red S to detect calcium deposits. For adipogenic differentiation, cells were cultured in the adipogenic differentiation medium (Cyagen Biosciences) for 21 days when the cells reach 100 % confluent or post confluent, and stained with Oil red O solution to identify the presence of lipid-rich vacuoles.

### Flow cytometry

To identify the isolated fibroblast-like cells, flow cytometric analysis was applied for detecting their immunophenotype. In brief, cells at passage 4 were trypsinized and cell suspension containing 1 × 10^5^ cells were incubated with the monoclonal antibodies against CD14, CD34, and CD45 (FITC-conjugated); CD29, CD44, CD90, and CD105 (PE-conjugated) (BD Biosciences, San Jose, CA, USA) for 30 min at 4 °C in the dark. After washing, the labeled cells were resuspended in PBS and analyzed on a FACSCanto II flow cytometer (BD Biosciences, Sparks, MD, USA). Isotype-matched antibodies with the corresponding fluorescent labeling were used for measuring the nonspecific background signals.

### Generation of conditioned medium

GC-MSCs were plated in 35 cm^2^ flasks and grown in L-DMEM with 15 % FBS. Once reaching 80 % confluence, the cells were washed with PBS and re-incubated with 4 ml complete medium at 37 °C with 5 % CO_2_. After 48 hrs, the conditioned medium (CM) was collected, spun to remove cellular debris (1200 rpm for 10 min) and passed through a 0.22 μm filter. Aliquots were frozen and stored at -140 ˚C until use. GCN-MSC-, BM-MSC-, or gastric caner cell-derived CM was generated in a similar manner.

### MTT assay

BGC-823 or MKN-28 cells were plated into a 96-well plate at 3 × 10^3^ cells/well and cultured overnight. Afterwards, the medium was aspirated off and cells were treated with 10 % CM from GC-MSCs, GCN-MSCs, or BM-MSCs, respectively. After incubation for 48 hrs, MTT solution (5 mg/ml) was added into each well and incubated with the cells at 37 °C for 4 hrs. Then the solution was discarded and 150 μl dimethyl sulfoxide (DMSO) was added into the remaining cells for dissolving formazan crystals. The optical density (OD) at 490 nm was measured by a microplate reader (Benchmark, Bio-Rad Laboratories, Hercules, CA, USA), and all the experiments were performed in triplicate.

### Colony forming units assay

For colony forming units (CFU) assay, BGC-823, or MKN-28 cells were plated in duplicate at 200 cells/well in a 24-well plate. To evaluate the effect of paracrine factors, tumor cells were incubated in 10 % CM from GC-MSCs, GCN-MSCs, or BM-MSCs, respectively. Cultures were grown at 37 °C in a humidified incubator with 5 % CO_2_ for 10 days. Afterwards, the adherent cells were washed with PBS, fixed with 4 % paraformaldehyde and stained with 0.5 % Crystal violet for 10 min. Cluster of ≥ 50 cells were considered as colonies.

### Transwell migration assay

BGC-823 or MKN-28 cells were plated at 4 × 10^4^ cells/well in 8.0-mm pore sized 24-well Transwell inserts with serum-free L-DMEM. Control medium or 10 % CM from GC-MSCs, GCN-MSCs, or BM-MSCs was added into the lower chamber of transwell dishes and incubated at 37 °C for 16 hrs. After cell migration, the inserts were discarded, and upper side of the filter was swabbed to remove the nonmigratory cells. The filters were then fixed in 4 % paraformaldehyde and stained with 0.5 % Crystal violet for 20 min. Microscopic examination was performed and 8 randomly non-overlapping high-power fields (HPFs, × 200) were selected to count the stained migrated cells. All the experiments in each group were performed in triplicate.

### RT-PCR analysis

To investigate the pro-angiogenic effect of gastric cancer cells affected by MSCs, BGC-823, and MKN-28 cells were treated with 10 % CM from GC-MSCs, GCN-MSCs, or BM-MSCs, respectively. Conversely, the there different types of MSCs were also exposed to 10 % CM collected from BGC-823 and MKN-28 cells. Cells cultured in control medium served as the controls. After 72 hrs culture, cells in each group were harvested in TRIzol reagent (Life Technologies, Invitrogen, Carlsbad, CA, USA), and total RNA was extracted. A NanoDrop-2000 spectrophotometer (ThermoScientific, Waltham, MA, USA) was used to determine the yield and purity of total RNA. Following the manufacturer’s instructions, 3 μg RNA was processed for cDNA synthesis with Superscript II reverse transcriptase (Roche Diagnostics, Indianapolis, IN, USA) in a 40 μl reaction volume. PCR was performed in a reaction mixture of 25 μl volume containing 12.5 μl Premix Ex Taq (Takara, Shiga, Japan), 1 μl each primer (10 μM) and 2 μl cDNA samples. Primers of human VEGF, MIP-2, TGF-β1, IL-6, IL-8, and β-actin were designed using the Primer Software as shown in Table 1, and amplification was performed on the Veriti 96 Well Thermal Cycler (Applied Biosystems, Foster City, CA, USA). The expression levels of pro-angiogenic factors in BGC-823 and MKN-28 cells exposed to GC-MSC-CM pretreated with anti-IL-8 Ab were also analyzed by RT-PCR.

**Table 1:** Primer sequences for the amplification of target genes

Genes	Accession number	Primer sequence (5′–3′)	Product size (bp)	Annealing temperature (°C)
VEGF	NM_001025366	For: 5′-TTGCCTTGCTGCTCTACCTC-3′	198	60
		Rev: 5′-CACAGGATGGCTTGAAGATG-3′		
MIP-2	NM_002089	For: 5′-AACCGAAGTCATAGCCACAC-3′	150	59
		Rev: 5′-CAGGAACAGCCACCAATAAG-3′		
TGF-β1	NM_000660	For: 5′-CACGTGGAGCTGTACCAGAA-3′	114	61
		Rev: 5′-CACAACTCCGGTGACATCAA-3′		
IL-6	NM_000600	For: 5′-GAGGAGACTTGCCTGGTGAA-3′	267	60
		Rev: 5′-GCGCAGAATGAGATGAGTTG-3′		
IL-8	NM_000584	For: 5′-ACCGGAAGGAACCATCTCAC-3′	822	61
		Rev: 5′-GTGGATCCTGGCTAGCAGAC-3′		
β-actin	NM_001101	For: 5′-TGGACTTCGAGCAAGAGATG-3′	207	60
		Rev: 5′-GGATGTCCACGTCACACTTC-3′		

### Tube formation assay

Tube formation assay was further performed to evaluate the regulating effect of GC-MSCs on tumor angiogenesis of BGC-823 and MKN-28 cells. The procedure was carried out according to a previously published method [14] with slight modifications. Briefly, matrigel (50 μl/well, BD Pharmingen, San Diego, CA, USA) was added into a 96-well plate and incubated for 1 hr at 37 °C for matrix formation. After that, human umbilical vein endothelial cells (HUVECs) were seeded onto the gel at a density of 1.5 × 10^4^ cells/well in α-DMEM with 10 % FBS, 10 % CM from GC-MSCs, 10 % CM from cancer cells, and 10 % CM from co-culture of GC-MSCs and cancer cells, respectively. After incubation for 8 hrs, the cells were visualized, and photographed by an inverted microscope to evaluate the tube-like structure formation (× 100).

### Luminex immunoassay

To indentify the key factors mediating the tumor-promoting effect of GC-MSCs, we collected cell supernatants of GC-MSCs, GCN-MSCs, and BM-MSCs at 48 hrs of cell cultivation and quantified the concentrations of 10 cytokines/chemokines by Luminex immunoassay. Moreover, the levels of cytokines/chemokines secreted by BGC-823 cells treated with 10 % CM from MSCs or MSCs exposed to 10 % BGC-823-CM were detected to confirm the effect of interaction between gastric cancer cells and GC-MSCs on tumor angiogenesis and progression. To make data comparable, the supernatants were harvested in a way that the concentration and culture time were coincident.

For all supernatant samples, an aliquot was immediately stored at -140 °C and subsequently thawed for the multiplexed bead array immunoassay based on a Bio-Plex 200 platform (Bio-Rad Laboratories). Custom-designed MILLIPLEXH human cytokine/chemokine 96-well plate assays (Cat. # 96-HBK1, Millipore Corporation, Billerica, USA) were performed according to the manufacturer’s protocol.

### Neutralization assay by IL-8 blockade

To evaluate the effect of IL-8 on gastric cancer progression prompted by GC-MSCs, neutralization assay was performed in our study. Briefly, BGC-823, or MKN-28 cells were treated with 10 % GC-MSC-CM alone or together with a human IL-8 neutralizing antibody (R&D Systems, Minneapolis, MN, USA) at a concentration of 10 μg/ml. Mixtures of GC-MSC-CM and anti-IL-8 Ab were incubated at 4 °C overnight before being added to BGC-823 or MKN-28 cells. The proliferation, migration, and pro-angiogenesis abilities of gastric cancer cells were assessed after IL-8 neutralizing antibody treatment.

### Western blot

Cell extracts were prepared with RIPA buffer supplemented with complete protease inhibitors. Aliquots of proteins were separated in 12 % SDS-PAGE and transferred onto PVDF membrane, which was blocked with 5 % (w/v) blotting grade milk for 1 hr. Membranes were then incubated with the primary antibodies overnight at 4 °C. Sources of the primary antibodies were: anti-p-Akt, anti-Akt, anti-p-p44/42 MAPK (Erk1/2), and anti-p44/42 MAPK (Erk1/2) (Cell Signaling Technology, Beverly, MA, USA), and anti-β-actin (Santa Cruz Biotechnology, Santa Cruz, CA, USA).

### Statistical analysis

Results were expressed as mean ± SEM. Data were analyzed and plotted with GraphPad Prism software 6.0 (GraphPad Software, La Jolla, CA, USA). Statistic analysis was performed by non-parametric Mann-Whitney *U* test using SPSS 16.0 statistical software, and *P*-value < 0.05 was considered statistically significant.

## Results

### Isolation and characterization of GC-MSCs

**Fig. 1:**
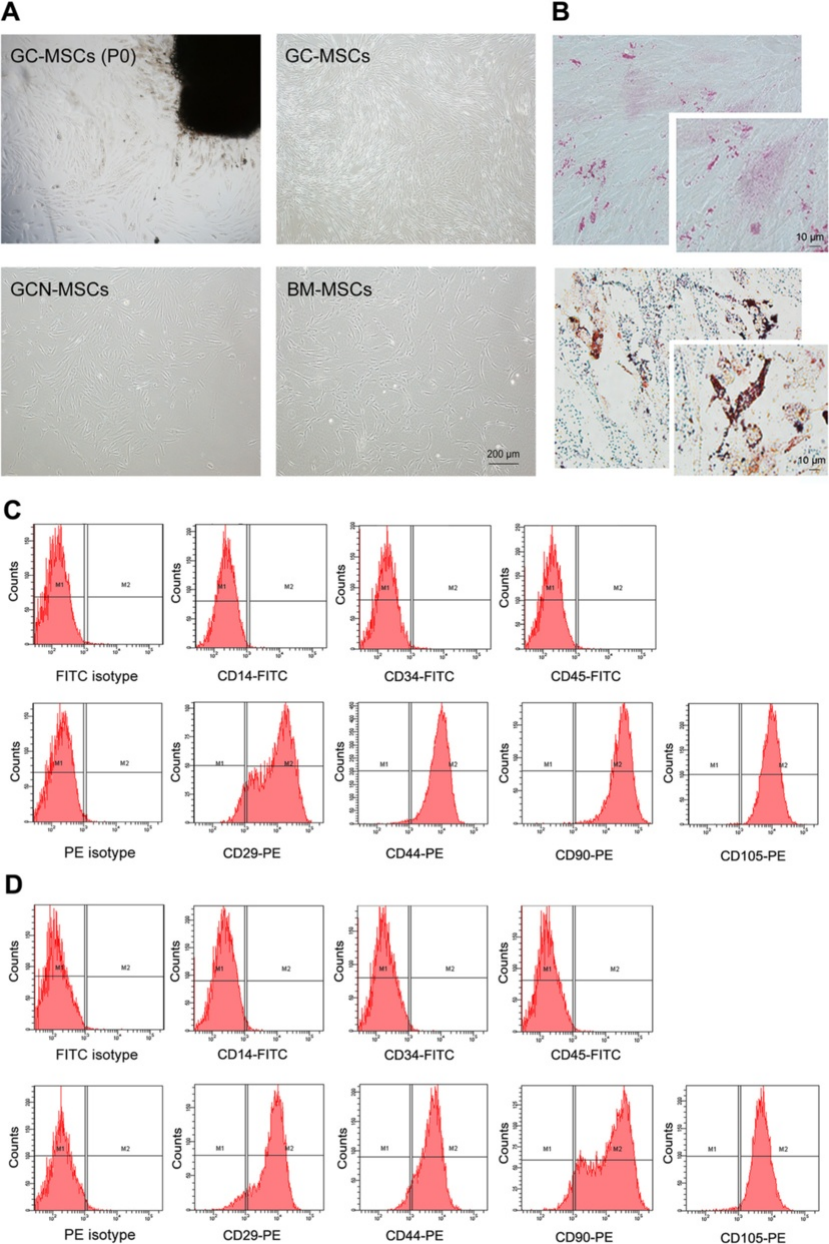
Characterization of human gastric cancer-derived MSCs. (**A**) Spindle-shaped cells migrated from gastric cancer tissues after 7–10 days of primary culture (upper left) and fibroblast-like cells appeared at passage 4 of GC-MSCs culture (upper right) with the morphology similar to GCN-MSCs (lower left) or BM-MSCs (lower right) (×40). (**B**) Representative photographs of GC-MSCs differentiated into mineralizing cells with alizarin red S staining (upper) and adipogenic cells with Oil red O staining (lower) (×200, ×400). (**C**) Surface antigens expressed on GC-MSCs analyzed by flow cytometry. (**D**) Surface antigens expressed on GCN-MSCs analyzed by flow cytometry

After 7–14 days of primary culture, a small population of fibroblast-like cells had migrated from gastric cancer tissues and expended *in vitro* (Fig. 1A). After plated into flasks, the cells exhibited spindle-shaped morphology, which were similar to GCN-MSCs or BM-MSCs (Fig.1A). Moreover, the pluripotent differentiation potential of GC-MSCs was evaluated *in vitro*. After induction for 14 days, alizarin red S staining revealed that mineralized extracellular matrix had generated in the cytoplasm of GC-MSCs (Fig. 1B), suggesting osteoblastic differentiation potential of GC-MSCs. On the other hand, the cells were shown to be filled with lipid-rich vacuoles by Oil red O staining after adipogenic induction (Fig. 1B). Furthermore, flow cytometry analysis conveyed that both GC-MSCs and GCN-MSCs were positive for CD29, CD44, CD90, and CD105, but negative for CD14, CD34, and CD45 (Fig. 1C, D), which displayed the characteristic surface markers of MSCs. Together, these data identifies the MSC-like characteristics of GC-MSCs.

### GC-MSCs facilitate the proliferation of gastric cancer cells more potently than GCN-MSCs or BM-MSCs

**Fig. 2:**
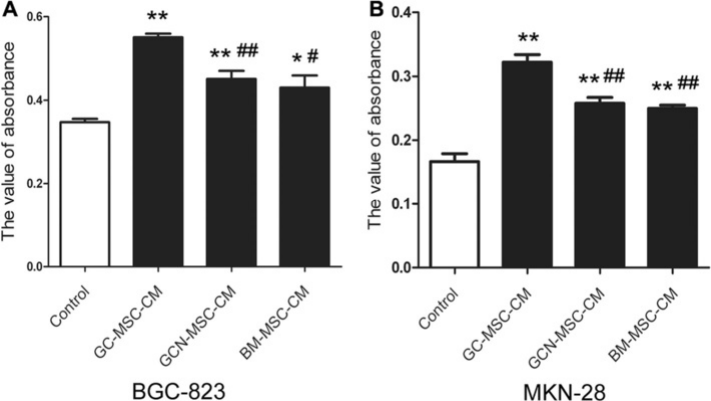
Effect of GC-MSCs on gastric cancer cell proliferation analyzed by MTT assay. (**A**) Viability of BGC-823 cells cultured with 10 % CM from GC-MSCs, GCN-MSCs, or BM-MSCs. (**B**) Viability of MKN-28 cells cultured with 10 % CM from GC-MSCs, GCN-MSCs, or BM-MSCs. *P < 0.05, **P < 0.01: compared with the control group; ^#^ *P* < 0.05, ^##^ *P* < 0.01: compared with the GC-MSC-CM treated group

We performed MTT assay and CFU assay to investigate whether GC-MSCs could promote gastric cancer cell growth and to compare the tumor-promoting activity of GC-MSCs with MSCs from non-malignant tissues. As indicated by the results of MTT assay, both BGC-823, and MKN-28 cells showed significantly increases in cell proliferation when cultured in 10 % CM from GC-MSCs, GCN-MSCs, or BM-MSCs compared with the controls (*P* < 0.05) (Fig. 2). Moreover, GC-MSCs displayed a significantly higher potential in promoting gastric caner cell proliferation than GCN-MSCs or BM-MSCs (*P* < 0.05) (Fig. 2).

**Fig. 3:**
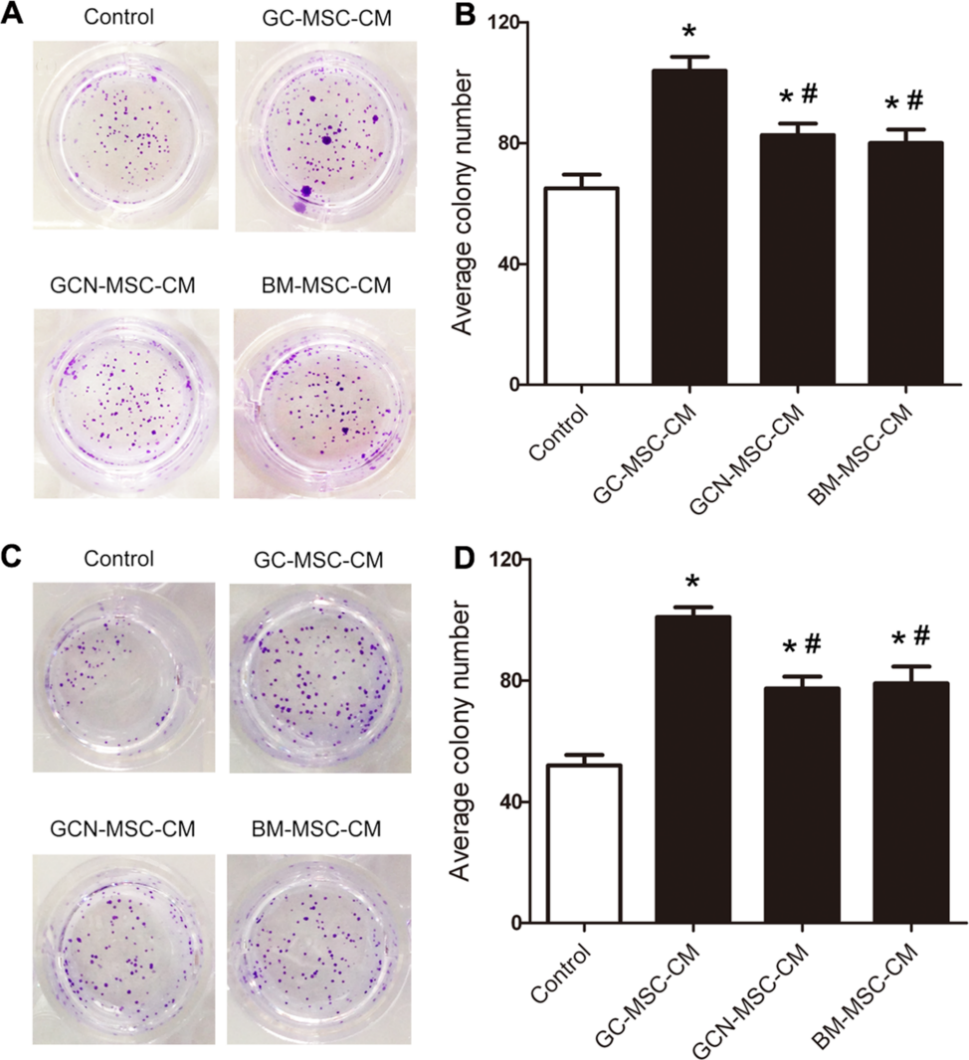
Effect of GC-MSCs on gastric cancer cell proliferation assessed by CFU assay. (**A**) Representative images of BGC-823 cell colony formation treated with 10 % CM from GC-MSCs, GCN-MSCs, or BM-MSCs. (**B**) Average colony numbers ± SD were plotted of BGC-823 cells in different groups (*n* = 3). (**C**) Representative images of MKN-28 cell colony formation treated with 10 % CM from GC-MSCs, GCN-MSCs, or BM-MSCs. (**D**) Average colony numbers ± SD were plotted of MKN-28 cells in different groups (*n* = 3). *P < 0.05: compared with the control group; ^#^ *P* < 0.05: compared with the GC-MSC-CM treated group

In accordance with MTT assay, CFU analysis revealed that gastric cancer cells treated with 10 % CM from GC-MSCs, GCN-MSCs, or BM-MSCs grew faster than the controls (Fig. 3). Furthermore, 10 % GC-MSC-CM treated BGC-823 and MKN-28 cells both showed higher average colony numbers (*P* < 0.05) and formed lager colonies than 10 % GCN-MSC-CM or BM-MSC-CM treated group (Fig. 3), suggesting a markedly potent tumor-promoting effect of GC-MSCs. Together, these results suggest that MSCs isolated from gastric cancer tissues could prompt gastric cancer cell proliferation more potently than GCN-MSCs or BM-MSCs *in vitro*.

### GC-MSCs promote the migration of gastric cancer cells more efficiently than GCN-MSCs or BM-MSCs

**Fig. 4:**
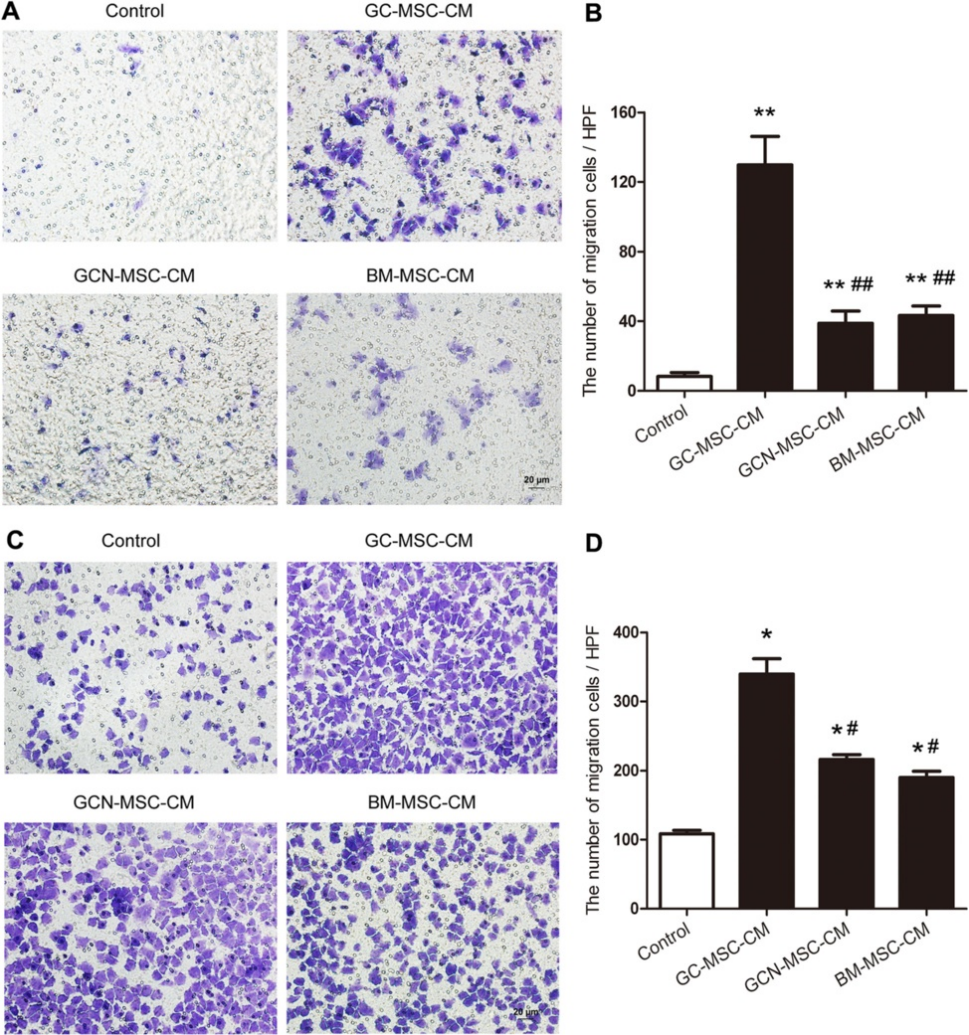
Effect of GC-MSCs on gastric cancer cell migration analyzed in a transwell system. (**A**) Representative images of migrated BGC-823 cells treated with 10 % CM from GC-MSCs, GCN-MSCs, or BM-MSCs (×200). (**B**) The numbers of migrated BGC-823 cells ± SD were plotted in different groups (*n* = 3). (**C**) Representative images of migrated MKN-28 cells treated with 10 % CM from GC-MSCs, GCN-MSCs, or BM-MSCs (×200). (**D**) The numbers of migrated MKN-28 cells ± SD were plotted in different groups (*n* = 3). *P < 0.05, **P < 0.01: compared with the control group; ^#^ *P* < 0.05, ^##^ *P* < 0.01: compared with the GC-MSC-CM treated group

We exploited transwell migration assay to investigate the regulating effect of GC-MSCs on gastric cancer cell migration. The number of viable cancer cells migrating into the lower chamber was the highest in 10 % GC-MSC-CM treated group than the other groups (Fig. 4). Although 10 % GCN-MSC-CM or BM-MSC-CM also showed significantly improved recruitment effect on BGC-823 and MKN-28 cells, a robust migration of tumor cells was observed upon exposure to 10 % GC-MSC-CM and the number of tumor cells attracted by 10 % GC-MSC-CM was strikingly higher than that by 10 % GCN-MSC-CM or BM-MSC-CM (*P* < 0.05) (Fig.4). Thus, these data demonstrate that gastric cancer cells may be preferentially recruited by GC-MSCs resident in the tumor tissues.

### GC-MSCs exhibit higher ability of pro-angiogenesis than GCN-MSCs or BM-MSCs

**Fig. 5:**
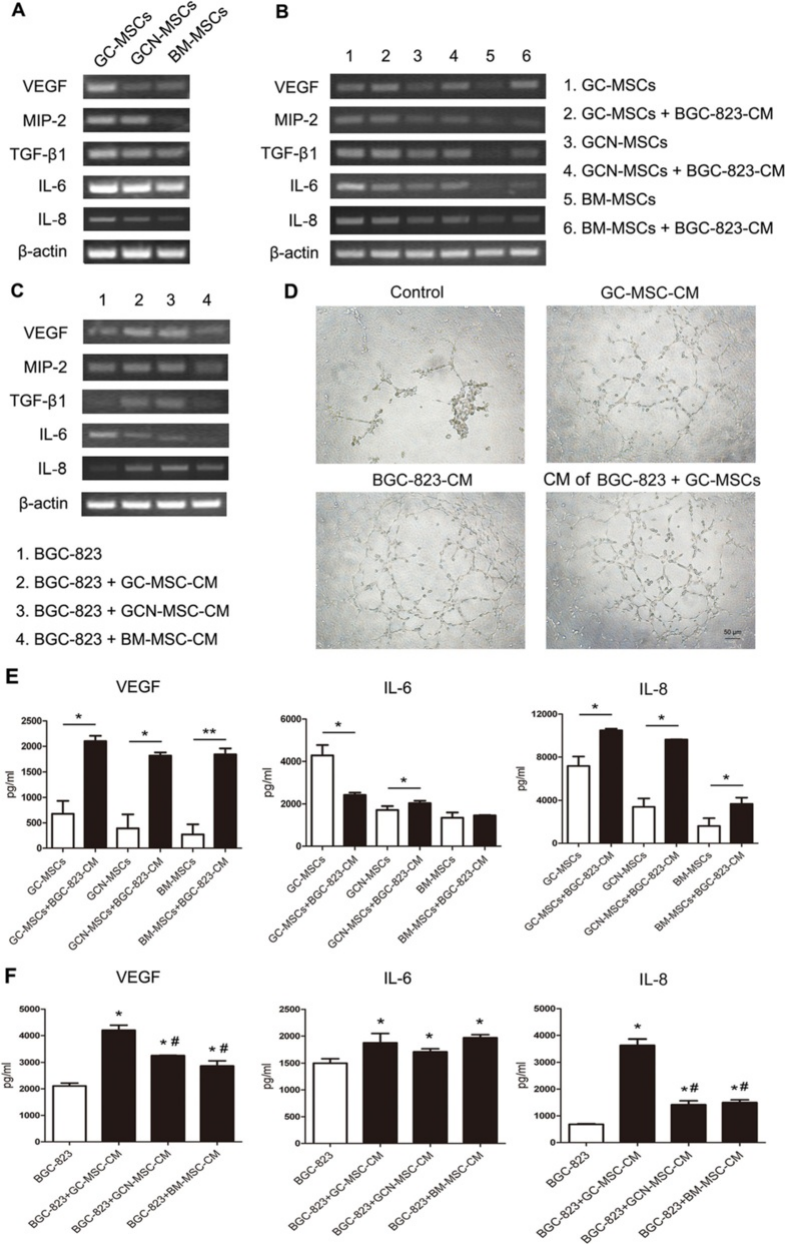
Regulating effect of interaction between BGC-823 cells and GC-MSCs on tumor angiogenesis. (**A**) RT-PCR analysis of VEGF, MIP-2, TGF-β1, IL-6, and IL-8 expression in GC-MSCs, GCN-MSCs, or BM-MSCs. (**B**) RT-PCR analysis of VEGF, MIP-2, TGF-β1, IL-6, and IL-8 expression in GC-MSCs, GCN-MSCs, or BM-MSCs after exposed to 10 % BGC-823-CM for 3 days. (**C**) RT-PCR analysis of VEGF, MIP-2, TGF-β1, IL-6, and IL-8 expression in BGC-823 cells after treated with 10 % CM from GC-MSCs, GCN-MSCs, or BM-MSCs for 3 days. (**D**) Representative photographs of HUVECs seeded on Matrigel for 8 hrs in culture medium (upper left) and in the presence of 10 % CM from GC-MSCs (upper right), BGC-823 cells (lower left), or co-cultured BGC-823 cells and GC-MSCs (lower right) (×100). (**E**) Luminex assay of VEGF, IL-6, and IL-8 secretion in the supernatant of GC-MSCs, GCN-MSCs, or BM-MSCs with 10 % BGC-823-CM treatment for 3 days. (**F**) Luminex assay of VEGF, IL-6, and IL-8 secretion in the supernatant of BGC-823 cells after exposed to 10 % CM from GC-MSCs, GCN-MSCs, or BM-MSCs for 3 days. *P < 0.05, **P < 0.01: compared with the control group; ^#^ *P* < 0.05: compared with the GC-MSC-CM treated group

As one of the key components in TME, MSCs have been found to play a critical role in tumor vascularization. In this study, we firstly investigated, and compared the mRNA expressions of pro-angiogenic factors in GC-MSCs with MSCs from non-malignant tissues. As shown in Fig. 5A, mRNA levels of VEGF, MIP-2, TGF-β1, IL-6, and IL-8 were the highest in GC-MSCs than those in GCN-MSCs or BM-MSCs, suggesting a more potent vascularization activity of GC-MSCs.

**Fig. 6:**
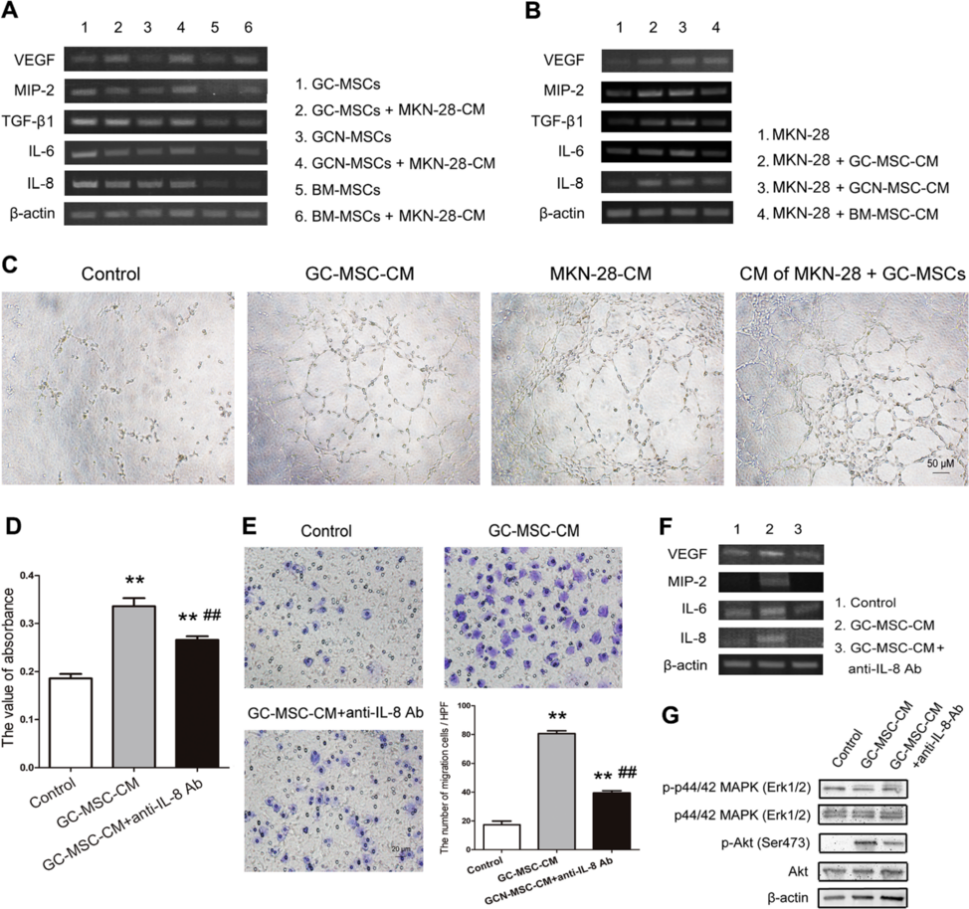
Pro-angiogenesis and tumor-promoting effect of GC-MSC-secreted IL-8 on MKN-28 cells. (**A**) RT-PCR analysis of VEGF, MIP-2, TGF-β1, IL-6, and IL-8 expression in GC-MSCs, GCN-MSCs, or BM-MSCs after exposed to 10 % MKN-28-CM for 3 days. (**B**) RT-PCR analysis of VEGF, MIP-2, TGF-β1, IL-6, and IL-8 expression in MKN-28 cells after treated with 10 % CM from GC-MSCs, GCN-MSCs, or BM-MSCs for 3 days. (**C**) Representative photographs of HUVECs seeded on Matrigel for 8 hrs in culture medium and in the presence of 10 % CM from GC-MSCs, MKN-28 cells, or co-cultured MKN-28 cells and GC-MSCs (×100). (**D**) Viability of MKN-28 cells cultured in 10 % GC-MSC-CM treated with or without anti-IL-8 antibody by MTT assay. (**E**) Transwell migration assay of MKN-28 cells exposed to 10 % GC-MSC-CM with or without anti-IL-8 antibody treatment (×200). (**F**) Expression of VEGF, MIP-1, IL-6, and IL-8 in MKN-28 cells exposed to 10 % GC-MSC-CM with or without IL-8 blockade. (**G**) Western blot for protein levels of Akt, p-Akt, p44/42 MAPK (Erk1/2), and p-p44/42 MAPK (Erk1/2) in MKN-28 cells stimulated by 10 % GC-MSC-CM with or without IL-8 blockade

To investigate the effect of tumor cells on angiogenesis ability of GC-MSCs, we detected the expressions of pro-angiogenic factors by GC-MSCs, GCN-MSCs, or BM-MSCs after treated with 10 % BGC-823-CM or MKN-28-CM *in vitro*. The results of RT-PCR indicated that there were no appreciable increases in the expression of pro-angiogenic factors in GC-MSCs after exposed to 10 % CM of gastric cancer cells, although the expression of VEGF was slightly up-regulated. Conversely, mRNA levels of VEGF, MIP-2, TGF-β1, IL-6, and IL-8 were all significantly up-regulated in GCN-MSCs or BM-MSCs after 10 % BGC-823-CM or MKN-28-CM treatment (Fig. 5B and Fig. 6A), suggesting a converted progression of non-malignant MSCs by tumor cells. On the other hand, Luminex assay demonstrated that the secretions of pro-angiogenic factors VEGF and IL-8 were all significantly up-regulated in the supernatant of GC-MSCs, GCN-MSCs, or BM-MSCs after 10 % BGC-823-CM stimulation (*P* < 0.05) (Fig. 5E). Thus, the above results suggest that GC-MSCs might play an important role in tumor neovascularization and this ability may be converted by gastric cancer cells in a paracrine manner.

### GC-MSCs enhance pro-angiogenesis ability of gastric cancer cells more potently than GCN-MSCs or BM-MSCs

Given that GC-MSCs could potently enhance the proliferation and migration of gastric cancer cells, we further wondered whether they affected the pro-angiogenic ability of tumor cells, which is considered as a critical step for tumor progression. As shown in Fig. 5C, incubation with 10 % CM from GC-MSCs or GCN-MSCs dramatically increased the expressions of VEGF, MIP-2, TGF-β1, and IL-8 in BGC-823 cells compared with the control. The similar effect was also confirmed in MKN-28 cells (Fig. 6B). In addition, the results of Luminex immunoassay showed that significantly elevated levels of VEGF and IL-8 were detected in the supernatant of BGC-823 cells treated with 10 % GC-MSC-CM in comparison to the other groups (*P* < 0.05) (Fig. 5F), suggesting a more effective promoting role of GC-MSCs in tumor cell angiogenesis than GCN-MSCs or BM-MSCs.

On the other hand, the results of tube formation assay demonstrated that 10 % CM from gastric cancer cells, GC-MSCs, or co-cultured gastric cancer cells and GC-MSCs enhanced tube formation by HUVECs compared with the CM-free control (Fig. 5D and Fig. 6C). Furthermore, exposure to 10 % CM from co-cultured gastric cancer cells and GC-MSCs resulted in a more branched network than 10 % gastric cancer cell-CM or GC-MSC-CM alone (Fig. 5D and Fig. 6C), suggesting a dramatically enhanced ability of co-cultured cells to induce the formation of tube-like structure. Thus, our results indicate that GC-MSCs could largely enhance the ability of gastric cancer cells to prompt angiogenesis through up-regulation of the pro-angiogenic factors.

### IL-8 secretion is strikingly high in the supernatant of GC-MSCs

**Fig. 7:**
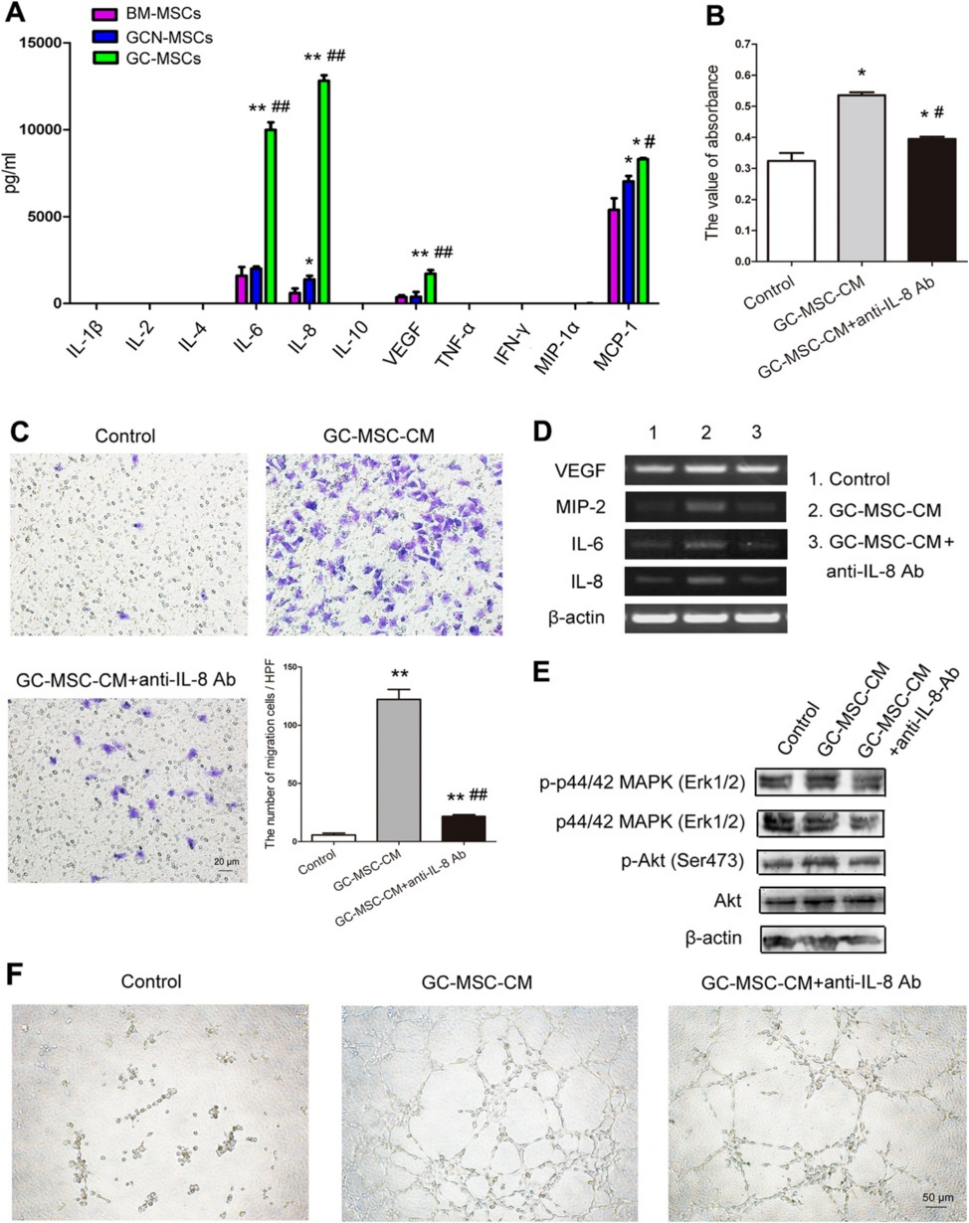
Tumor-promoting effect of GC-MSCs on BGC-823 cells is attenuated by the application of anti-IL-8 antibody. (**A**) Cytokine profile analysis of GC-MSCs, GCN-MSCs, or BM-MSCs by Luminex immunoassay. (**B**) Viability of BGC-823 cells cultured in 10 % GC-MSC-CM treated with or without anti-IL-8 antibody by MTT assay. (**C**) Transwell migration assay of BGC-823 cells exposed to 10 % GC-MSC-CM with or without anti-IL-8 antibody treatment (×200). (**D**) Expression of VEGF, MIP-1, IL-6, and IL-8 in BGC-823 cells exposed to 10 % GC-MSC-CM with or without IL-8 blockade. (**E**) Western blot for protein levels of Akt, p-Akt, p44/42 MAPK (Erk1/2), and p-p44/42 MAPK (Erk1/2) in BGC-823 cells stimulated by 10 % GC-MSC-CM with or without IL-8 blockade. (**F**) Representative photographs of capillary tube formation by HUVECs exposed to 10 % GC-MSC-CM with or without IL-8 blockade. (×100). *P < 0.05, **P < 0.01: compared with the control group; ^#^ *P* < 0.05, ^##^ *P* < 0.01: compared with the GC-MSC-CM treated group

Since GC-MSCs were demonstrated to affect proliferation, migration, and angiogenesis of gastric cancer in a paracrine manner, the key factors contributing to the tumor-promoting role of GC-MSCs were further analyzed in this study. Cytokines/chemokines including VEGF, MCP-1, IL-6, and IL-8 were detectable in the supernatant of GC-MSCs, GCN-MSCs, or BM-MSCs, whereas GC-MSCs elaborated a strikingly higher level of IL-8 secretion with significant difference from GCN-MSC-CM or BM-MSC-CM (*P* < 0.01) (Fig. 7A). Along these lines, we postulate that the tumor-promoting effect of GC-MSCs may be partly mediated by IL-8 secretion.

### Anti-IL-8 antibody attenuates the gastric cancer-promoting effect of GC-MSCs

Since IL-8 is reported to play a paramount role in tumor progression and strikingly high level of IL-8 was detected in the supernatant of GC-MSCs in our study, we wondered whether IL-8 mediated the tumor-promoting effect of GC-MSCs in gastric cancer. The results of MTT assay indicated that simultaneous treatment with 10 % GC-MSC-CM and anti-IL-8 antibody resulted in a significant decrease in the proliferation of BGC-823 or MKN-28 cells compared with 10 % GC-MSC-CM treated group (*P* < 0.05) (Fig. 7B and Fig. 6D). In addition, the number of BGC-823, or MKN-28 cells attracted by 10 % GC-MSC-CM was dramatically reduced by anti-IL-8 antibody (*P* < 0.01) (Fig. 7C and Fig. 6E). Furthermore, we investigated the role of IL-8 in gastric cancer angiogenesis promoted by GC-MSCs. As shown in Fig. 7D, increased expressions of VEGF, MIP-2, IL-6, and IL-8 in BGC-823 cells by 10 % GC-MSC-CM were abrogated in the presence of IL-8-specific neutralizing antibody. Similar results were also obtained in MKN-28 cells (Fig. 6F). Accordingly, anti-IL-8 blockade markedly attenuated the formation of tube-like structures by HUVECs after 10 % GC-MSC-CM stimulation (Fig. 7F). In addition, Western blot analysis revealed that 10 % GC-MSC-CM also led to increased phosphorylation of Akt or p44/42 MAPK (Erk1/2) in gastric cancer cells, which was partly abrogated by IL-8-specific neutralizing antibody treatment (Fig. 7E and Fig. 6G). Thus, our data indicate that the tumor-promoting effect of GC-MSCs is partly blocked by IL-8-specific neutralizing antibody, which suggests a critical role of IL-8 in gastric caner progression mediated by GC-MSCs.

## Discussion

During the past decades, correlation between MSCs, and tumor has drawn a lot of attention and been reported to play a critical role in tumor progression. Upon tumorigenesis, non-cancerous tissues-derived MSCs such as BM-MSCs are recruited into tumor mass and incorporate into the stromal microenvironment [25, 26]. After continuously exposed to inflammatory factors and other stromal cells in local tumor tissues, BM-MSCs may be instructed to adopt some new features and become tumor-resident MSCs [27, 28]. Although considerable studies have performed to investigate the correlation between non-cancerous tissues-derived MSCs and tumor [29–31], the detailed properties of converted tumor-resident MSCs and their role in tumor growth and progression merit further investigation.

Gastric cancer is one of the most frequent malignant tumors, which has affected humans for millennia. Despite the improved prognosis, overall 5-year survival rates for patients of gastric cancer remain disappointing. For new therapeutic strategies development, an improved understanding of the mechanisms in gastric cancer growth and progression is urgently needed. In this study, we isolated resident MSCs from human gastric cancer tissues, which showed a multi-lineage differentiation potential and a heterogeneous immunophenotype with fibroblastic morphology. We studied the promoting effect of GC-MSCs on the proliferation, migration, and pro-angiogenic capabilities of gastric cancer cells *in vitro* and compared it with non-malignant tissue-derived GCN-MSCs and BM-MSCs. In addition, we further investigated the underlying mechanism involved in the tumor-promoting effect of GC-MSCs.

Firstly, we observed the influence of GC-MSCs in gastric cancer cell proliferation. The results showed that BGC-823 and MKN-28 cells were both stimulated to grow faster when incubated with 10 % GC-MSC-CM, which displayed a more potent tumor-promoting ability than GCN-MSC-CM or BM-MSC-CM. This suggests a pivotal role of gastric cancer-resident MSCs in tumor cell proliferation. In keeping with our results, Guangwen, and colleagues reported that mouse lymphoma-derived MSCs present a more potently effect of tumor growth-promotion than BM-MSCs or MSCs from other normal tissues such as skin [16]. Another study also conveyed that MSCs from human breast cancer tissues have certain increased effect on the growth of breast cancer *in vitro* [32]. Consequently, we investigated the effect of GC-MSCs on gastric cancer cell recruitment by a transwell migration assay. A more drastic promotion was observed in the migration of gastric cancer cells with 10 % GC-MSC-CM stimulation compared with 10 % GCN-MSC-CM or BM-MSC-CM treatment, suggesting a greater potential of GC-MSCs to promote gastric cancer metastasis.

Furthermore, the pro-angiogenic role of GC-MSCs has drawn much interest in the present study, which may be involved in gastric cancer growth and metastasis. Ting and colleagues found that the crosstalk between tumor cells and BM-MSCs could increase the expression of pro-angiogenic factors and thereby promote growth and angiogenesis of breast and prostate tumors [14]. Another report proposed that MSC-secreted IL-6 may enrich the pro-angiogenic factors secreted by cancer cells to increase angiogenesis and tumor growth, and targeting this interaction may lead to novel therapeutic and preventive strategies [33]. In our study, GC-MSCs expressed higher levels of VEGF, MIP-2, TGF-β1, IL-6, and IL-8 than GCN-MSCs or BM-MSCs did, suggesting a more potent role of GC-MSCs in tumor angiogenesis. Consequently, we investigated the effect of gastric cancer cell-derived CM on the pro-angiogenic ability of GC-MSCs and observed an appreciable increase of VEGF both in mRNA and protein levels. Moreover, the expressions of VEGF, MIP-2, TGF-β1, IL-6, and IL-8 were all up-regulated in GCN-MSCs and BM-MSCs by 10 % BGC-823-CM or MKN-28-CM stimulation, suggesting a converted progression suffered by MSCs from non-malignant tissues by tumor cells. On the other hand, BGC-823, or MKN-28 cells exposed to 10 % GC-MSC-CM presented appreciable increase in pro-angiogenic ability, which may be associated with the promotions of growth and metastasis in gastric cancer.

How did GC-MSCs stimulate the proliferation, migration, and angiogenesis of gastric cancer cells? The underlying mechanism was further investigated in our study. According to the report by Yun and colleagues, IL-8 could stimulate VEGF production in BM-MSCs in part via the PI3K/Akt and MAPK/ERK signal pathways and administration of IL-8 treated BM-MSCs increases angiogenesis after stroke [23]. Ko and colleagues reviewed that IL-8 induced by *H. pylori* displays a major role in gastric cancer development and progression, and may be indicative of poor prognosis [19]. However, the mechanism has not been thoroughly understood in the context of gastric cancer. Our results of Luminex assay conveyed that the level of IL-8 was strikingly high in the secretome of GC-MSCs in comparison to GCN-MSCs or BM-MSCs, suggesting a potential of IL-8 as the key mediator for tumor-promoting activity of GC-MSCs. Furthermore, we demonstrated that the improved abilities of proliferation, migration, and angiogenesis in gastric cancer cells regulated by GC-MSCs can be attenuated by IL-8 neutralizing antibody. Furthermore, we also observed that GC-MSCs increased the activations of Akt and p44/42 MAPK (Erk1/2) in gastric cancer cells and this effect could be partly abrogated in the presence of IL-8-specific neutralizing antibody, indicating that GC-MSC-secreted IL-8 promoted gastric cancer growth and progression through regulating the signal pathways related to tumor growth and angiogenesis. Thus, these observations imply that the promoting effect of GC-MSCs on gastric cancer growth and progression are attributed to their strikingly highly secretion of IL-8. Blocking the interaction between GC-MSCs and gastric cancer cells by anti-IL-8 antibodies may provide a novel therapeutic or preventive strategy.

However, there are potential limitations of our experimental designs, as only two gastric cancer cell lines were supplied in this study and further investigation is needed to better understand the mechanisms related to cancer-resident MSCs in other tumor models. In addition, the *in vivo* experiments with mice model should be performed in future for better understanding of the mechanism underlying the tumor-promoting activity of IL-8-secreted GC-MSCs.

## Conclusions

Our present study demonstrates that gastric cancer-resident MSCs have a potent ability in promoting the proliferation, migration, and pro-angiogenesis of gastric cancer cells. IL-8, which is largely secreted by GC-MSCs, partly contributes to the tumor-promoting effect of GC-MSCs. In conclusion, we propose a new mechanism involved in gastric cancer that MSCs resident in gastric cancer tissues play a paramount role in cancer angiogenesis and progression through the secretion of cytokine IL-8, which may provide a novel avenue for gastric cancer therapy.

## Abbreviations

BM-MSCs: Bone marrow-derived mesenchymal stem cells
CFU: Colony forming units
CM: Conditioned medium
DMSO: Dimethyl sulfoxide
ERK1/2: Extracellular signal-regulated kinase 1/2
FBS: Fetal bovine serum
FITC: Fluorescein isothiocyanate
GC-MSCs: Gastric cancer-derived mesenchymal stem cells
GCN-MSCs: Gastric cancer adjacent non-cancerous tissue-derived mesenchymal stem cells
HPFs: High-power fields
HUVECs: Human umbilical vein endothelial cells
IL: Interleukin
L-DMEM: Dulbecco’s modified Eagle’s medium with low glucose
MAPK: Mitogen-activated protein kinase
MIP-2: Macrophage inflammatory protein-2
MSCs: Mesenchymal stem cells
OD: Optical density
PE: Phycoerythrin
PI3K: Phosphatidylinositol–4,5-bisphosphate 3-kinase
TGF-β1: Transforming growth factor-β1
TME: Tumor microenvironment
VEGF: Vascular endothelial growth factor
